# Factors Associated with Shortening of Prehospital Delay among Patients with Acute Ischemic Stroke

**DOI:** 10.3390/jcm8101712

**Published:** 2019-10-17

**Authors:** Raúl Soto-Cámara, Josefa González-Santos, Jerónimo González-Bernal, Asunción Martín-Santidrian, Esther Cubo, José M Trejo-Gabriel-Galán

**Affiliations:** 1 Department of Health Sciences, University of Burgos, 09001 Burgos, Spain; 2 Emergency Medical Service, 09200 Burgos, Spain; 3 Neurology Department, University Hospital of Burgos, 09006 Burgos, Spain

**Keywords:** ischemic stroke, prehospital delay, time factors, admission delay, early arrival

## Abstract

Background: Despite recent advances in acute stroke care, only 1–8% of patients can receive reperfusion therapies, mainly because of prehospital delay (PHD). Objective: This study aimed to identify factors associated with PHD from the onset of acute stroke symptoms until arrival at the hospital. Methods: A cross-sectional study was conducted including all patients consecutively admitted with stroke symptoms to Burgos University Hospital (Burgos, Spain). Socio-demographic, clinical, behavioral, cognitive, and contextualized characteristics were recorded, and their possible associations with PHD were studied using univariate and multivariable regression analyses. Results: The median PHD of 322 patients was 138.50 min. The following factors decreased the PHD and time until reperfusion treatment where applicable: asking for help immediately after the onset of symptoms (OR 10.36; 95% confidence interval (CI) 4.47–23.99), onset of stroke during the daytime (OR 7.73; 95% CI 3.09–19.34) and the weekend (OR 2.64; 95% CI 1.19–5.85), occurrence of stroke outside the home (OR 7.09; 95% CI 1.97–25.55), using a prenotification system (OR 6.46; 95% CI 1.71–8.39), patient’s perception of being unable to control symptoms without assistance (OR 5.14; 95% CI 2.60–10.16), previous knowledge of stroke as a medical emergency (OR 3.20; 95% CI 1.38–7.40), call to emergency medical services as the first medical contact (OR 2.77; 95% CI 1.32–5.88), speech/language difficulties experienced by the patient (OR 2.21; 95% CI 1.16–4.36), and the identification of stroke symptoms by the patient (OR 1.98; 95% CI 1.03–3.82). Conclusions: The interval between the onset of symptoms and arrival at the hospital depends on certain contextual, cognitive, and behavioral factors, all of which should be considered when planning future public awareness campaigns.

## 1. Introduction

Stroke is one of the most frequent causes of mortality, dependency, and disability among adults worldwide, leading to high economic costs for the patients, their families, and health services [1,2,3].

Despite recent advances in acute stroke care that can improve clinical outcomes [4,5], only 1–8% of patients can reach the hospital for reperfusion therapies [6,7,8], which is far from the minimum expected to decrease the effect of stroke at the population level significantly [9]. The term “time is brain” emphasizes the importance of the time in nervous tissue damage in acute stroke. The interval between the onset of symptoms and the arrival at the hospital greatly influences the treatment efficacy and the final prognosis of the patient [10]. Most of the studies on prehospital delay (PHD) have focused on the identification of the socio-demographic, clinical, and contextual characteristics of patients who choose not to seek immediate treatment [11,12,13,14]. However, the PHD can also be affected by other behavioral and cognitive factors and features that have hardly been studied [15].

This study aimed to identify socio-demographic, clinical, behavioral, cognitive, and contextual factors that influence the early arrival of patients with acute stroke at the hospital.

## 2. Methods

This one-year cross-sectional prospective study, part of a larger project on PHD, stroke awareness, and treatment, was conducted at the Neurological Department, Burgos University Hospital, a large-scale tertiary care center in north Spain, with a well-established intravenous thrombolysis program and stroke unit. Emergency medical service (EMS) was provided by the regional Emergency Medical Dispatch Centers, which responded to the European phone number (112) and used validated and standardized protocols for stroke screening [10]. No public awareness campaigns on stroke took place before or during the study period.

The study included patients of both sexes, aged ≥18 years, who were consecutively admitted to the emergency department (ED) during the first year, within 12 h after the onset of acute stroke. The diagnosis of stroke was as per the definition of the World Health Organization, and cerebral infarction based on neuroimaging (computed tomography (CT) or magnetic resonance imaging) results was differentiated from intraparenchymal hemorrhage [10]. Exclusion criteria for patients included the following: in-hospital stroke, episodes in which diagnostic workup was inconclusive or showed probable causes other than stroke, time of symptom onset was unknown, inability to communicate data directly because of speech or cognitive deficits, no follow-up in the first hours of admission, and refusal to provide informed consent. In the case of recurrent strokes during the study period, only the first event was included. The data were obtained without any prespecified hypotheses, and the sample size was not previously determined.

Patients were informed of the purpose of the study and invited to participate. In case of acceptance, they were asked to sign the informed consent. Patients answered a structured questionnaire for 15–20 min in a face-to-face interview within 72 h of admission to minimize in-hospital stroke education and information loss. The medical records were reviewed to obtain complete data, confirmed by participants. The study was approved by the Institutional Review Board (IRB 1479).

The primary outcome was PHD, the time in minutes from the onset of symptoms to the arrival at the hospital. The time of stroke onset was the moment when the patient or a witness first noticed a neurological deficit. The time of arrival was the earliest time routinely documented in the electronic medical record of the ED. The PHD was divided into two subperiods: the initial part or decision delay (DD), i.e., the interval between the symptom onset and first contact to ask for help depending on the patient, and the final part or transport delay (TD), i.e., the interval from the request for assistance to arrival at the hospital. 

Five domains were evaluated: socio-demographic features, clinical characteristics, behavioral response to symptoms, cognitive response to symptoms, and the context in which the stroke occurred. Stroke severity was assessed during admission using the “National Institute of Health Stroke Scale” (NIHSS) [16], representing the condition as mild to moderate (≤16) or severe (>16); if no score was documented, it was calculated retrospectively by reviewing the chart of the patient’s documented neurologic examination on hospital arrival. The degree of disability was previously assessed using the “modified Rankin scale,” representing the condition as independent (≤2) and dependent (>2). The COPE-28 questionnaire, a situational version, was used to determine the type of coping strategy [17]. The level of self-perceived seriousness and anxiety was assessed using a five-point Likert scale. The previous knowledge of stroke was determined by at least two signs or symptoms and two risk factors; the three possible responses, of which only one was correct, were proposed to evaluate the response to a possible case. The date of onset was categorized into working days (Monday to Friday) and weekends (Saturday, Sunday, and official holidays); the time of onset was divided into morning (06:00–14:00), afternoon (14:00–22:00), and night (22:00–06:00). The terms rural and urban were defined as the region outside or within the boundary of the city where the hospital was located, respectively. The mode of transport was classified as an ambulance or others.

PHD was not normally distributed; therefore, for descriptive statistics, the median values and interquartile range (IQR) were calculated. The association between PHD and possible explanatory variables was assessed using either the Mann–Whitney U-test or the Kruskal–Wallis test. A forward stepwise multivariable regression analysis and binary logistic regression analysis adjusted by sex and age were performed to identify possible predictive factors for PHD greater or lesser than 210 or 360 min, in which those variables with a *p*-value of <0.20 in the univariate analysis or those considered particularly relevant in the literature were included. The cutoff of 210 and 360 min was selected as per the time window for intravenous thrombolytic therapy, assuming an additional hour of intra-hospital delay, which would in no case exceed 270 min and for mechanical thrombectomy without CT perfusion, respectively [10]. A *p*-value of <0.05 was considered statistically significant. The statistical analysis was performed using the SPSS statistical software package Version 24.0 (IBM SPSS Inc, Chicago, IL, USA).

## 3. Results

Of the 583 eligible patients, 261 were excluded for the following reasons: arrived 12 h after stroke onset (*n* = 136), the stroke onset time was unknown (*n* = 53), no ischemic stroke on final diagnosis (*n* = 18), in-hospital stroke (*n* = 21), recurrent stroke (*n* = 19), no possibility of follow-up (*n* = 8), and inability to communicate data (*n* = 6). The characteristics of this group were similar to the 322 patients included in the study with respect to age and gender. 

**Figure 1:**
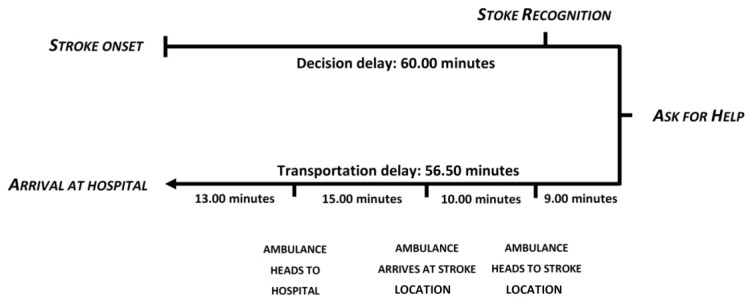
Time to arrival at the hospital segregated by landmarks.

For all patients, the median PHD was 138.50 (IQR 74–328) min. The median PHD was even shorter when the patient was transported by EMS to the hospital (median 128.00 min; IQR 71–268 min). If the patients were classified by PHD, more than three-fifths of patients (62.42%) arrived at the hospital in the first 210 min after symptom onset, 15.53% within 210–360 min, and one-fifth (22.05%) took more than 360 min. The median DD and TD were 60.00 (IQR 10–240) min and 56.50 (IQR 35–91) min, respectively. The median TD among the 221 patients who used the EMS was 47.00 (IQR 33–74) min, which was divided into four consecutive intervals: EMS activation delay (median 5.00 (IQR 5–28) min), EMS arrival delay (median 10.00 (IQR 7–16) min), EMS delay on scene (median 15.00 (IQR 10–23) min), and patient transport delay from the scene to hospital by EMS (median 13.00 (IQR 9–38) min) (Figure 1). Considering the maximum time limits [10], 33.23% of patients had a DD of <15 min, 36.64% had a TD of <45 min, and 62.42% had a PHD of <210 min. 

Table 1, Table 2, Table 3, Table 4 and Table 5 show the frequencies and the univariate analysis of PHD stratified by the socio-demographic, clinical, behavioral, cognitive, and contextual characteristics of the patients. No socio-demographic or clinical factor, except speech/language alteration and headache, had a significant effect on the median time from the symptom onset to hospital admission. However, most of the factors in the cognitive or contextual domains were associated with significant differences in median PHD. A shorter time interval between symptom onset and arrival at the hospital was associated with the following: asking for help as the first response after the onset of symptoms, activating EMS as the first medical contact, attributing symptoms to a possible stroke, self-perceiving the situation as serious, high anxiety level, previous knowledge of stroke, knowledge of risk factors or mode of action, being accompanied, symptom onset during the daytime (06:00–22:00 h) or in an urban area, arriving at the hospital in an ambulance, or activating the prehospital stroke code. However, the longer time interval was associated with the following: thinking that the situation could be self-managed or symptoms would improve spontaneously and being alone at home.

In the multivariable regression analysis, the following factors were significantly associated with a PHD of ≤210 min: asking for help immediately after symptom onset by the patient (OR 10.36; 95% confidence interval (CI) 4.47–23.99), stroke onset during the daytime (OR 7.73; 95% CI 3.09–19.34), using a prenotification system (stroke code; OR 4.54; 95% CI 1.77–11.64), patient’s perception of controlling symptoms without any assistance (OR 4.14; 95% CI 1.70–10.07), stroke occurrence outside the home (OR 3.03; 95% CI 1.22–7.55), calling EMS as the first medical contact (OR 2.77; 95% CI 1.32–5.88), and patient experienced speech/language difficulties (OR 2.21; 95% CI 1.16–4.36). When the repeated analysis was performed with early arrival defined as a PHD of ≤360 min, several results were consistent with those obtained for a PHD of ≤210 min. The previous knowledge of stroke as a medical emergency (OR 3.20; 95% CI 1.38–8.40), stroke onset during the weekends (OR 2.64; 95% CI 1.59–2.85), and identification of stroke symptoms by the patients (OR 1.98; 95% CI 1.03–3.82) were all associated with an increased likelihood of arriving at the hospital with a PHD of ≤360 min (Table 6). 

## 4. Discussion

The obtained median PHD after the onset of symptoms was lesser than that obtained by other studies [12,15,18,19,20]. No differences were observed between the median DD and TD. The cutoff time used in some studies for patients to reach hospital was 24 or 48 h or other periods [11,12,21,22]; however, we selected 12 has the cutoff time, as the patients are more likely to have thrombolysis or thrombectomy [23]. Therefore, the median delay data must be interpreted with caution. During the first 12 h, three-times more stroke patients arrived at the hospital when considered up to seven days, which is 14-fold longer interval.

Treating stroke early can reduce in-hospital delay and TD, which is not dependent on the patient, but on motivated professionals [24]. Moreover, earlier treatment can be obtained by improving the DD, the first link in the "stroke chain of survival." However, reducing DD is most difficult because patients with variable knowledge of their disease, who may suffer an unexpected stroke, can themselves reduce their ability to recognize it and request assistance [25].

Socio-demographic and clinical factors are nonmodifiable and have been previously reported to be associated with PHD, although conflicting and inconclusive results have been obtained in some studies [15,18,20,21,22]. In this study, speech/language disorder was the only factor independently associated with a PHD of ≤210 min (OR 2.21; 95% CI 1.16–4.36). Aphasia may be one of the most alarming symptoms and, therefore, may favor an earlier request for help, resulting in a shorter delay [13,18,26,27].

Behavioral and cognitive factors are associated with the patients and therefore can prolong or reduce the PHD. These factors have not been studied previously in association with stroke delay and therefore require further examination. 

Three-fifths of patients asked for help on the onset of symptoms in the first hour. The time to seek medical attention represented 51.50% of the PHD, similar to other studies [12,18]. If the early request for help was considered (DD <15 min) [12], only one-third of patients arrived at the hospital on time for thrombolysis. The likelihood of reaching hospital with a PHD of < 210 min was 10-times higher in patients who asked for help (OR 10.36; 95% CI 4.47–23.99), increasing to 17-fold if the patient did so within the first 15 min. The three main factors influencing the decision to seek help were the following: identifying the symptoms (*p* < 0.001), maintaining a sense of normality (*p* < 0.001), and the presence and influence of another person (*p* < 0.001); these results are consistent with those of previous studies [28]. In addition, stroke symptoms affect a patient’s ability to seek help; therefore, a longer delay in PHD may be noted for a considerable number of patients. Consistent with clinical practice, when the EMS was the first medical contact after symptom onset, the likelihood of arriving on time for thrombolysis was higher (OR 2.77; 95% CI 1.32–5.88), reducing the intra-hospital time by advancing some actions. Patients who activated the EMS had more serious stroke (*p* < 0.001). Moreover, 44.41% patients called EMS as the first medical contact, which is consistent with other studies [18,22,29], with 71.33% arriving within 210 min and 83.92% within 360 min. The lack of knowledge of the role of EMS in acute stroke may be a cause for its low use. Reaching the hospital by ambulance was also associated with a shorter PHD; however, its influence decreased after analyzing other factors. This difference may be because of the previous contact with EMS in two-thirds of patients transported by ambulance to the hospital. Therefore, despite using any means of transport, PHD was greatly influenced by the first medical contact, thus increasing the use of ambulance to reach the hospital.

Patients’ interpretation of symptoms was also crucial: when they thought the situation could not be self-managed, the PHD decreased to 253 min (OR 5.14; 95% CI 2.60–10.16). The PHD of patients with a generally proactive attitude was not shorter because they believed they could control the situation without any assistance or the symptoms would spontaneously improve [15,20,29,30]. It is unlikely that stroke-induced anosognosia is responsible for underestimating the disease because the right hemisphere strokes were not associated with a higher PHD (*p* = 0.859). Patients who recognized initial symptoms as stroke arrived at the hospital on time for thrombectomy without advanced neuroimaging compared with those who attributed the symptoms to other pathologies (OR 1.98; 95% CI 1.03–3.82). However, only 73.17% of patients recognized their symptoms as a stroke and went to the hospital or called the EMS. In line with several other studies, having had a stroke or similar symptoms was associated with better recognition of the acute event [18,22]. Internalizing how the patients perceive a stroke can improve its detection. Despite patients having knowledge of at least two symptoms or two risk factors in more than half of all cases, only 25.47% were able to identify the symptoms during its onset. Previous knowledge of responding after a stroke was associated with on-time arrival at the hospital for thrombectomy without advanced neuroimaging (OR 3.20; 95% CI 1.38–7.40). When the patients having prior experience of stroke were asked about what they had done previously on the onset of symptoms, 77 patients (78.57%) reported having gone to the hospital directly or having called the EMS (*p* = 0.038) [21,22,31]. Despite having a low level of previous knowledge (34.78%), the findings highlight the ability of patients to translate the knowledge automatically into action [32]. A history of previous strokes can improve knowledge, but not behavior, indicating the complexity of the challenge. The awareness of stroke symptoms and risk factors can decrease the PHD; however, it is not enough because its influence disappears when analyzed together with other factors. More use of existing devices that facilitate the request for assistance or novel ones that detect stroke and automatically issue an alert can reduce PHD.

Other factors influence the PHD depending on how and when the stroke occurred. In line with other studies, an association between stroke occurring outside the home and early arrival at the hospital was observed (OR 3.03; 95% CI 1.22–7.55; OR 7.09; 95% CI 1.95–25.55). When the stroke occurs in public places, symptom onset is more likely witnessed by family members, friends, or bystanders who often take a more active approach and request medical assistance quickly [33]. Patients having a stroke during the daytime (06:00–22:00) were admitted earlier than those having one during the nighttime (22:00–06:00) (OR 7.73; 95% CI 3.09–19.34) because it is difficult to recognize at night at home and patients often wait to recover spontaneously [26,27,34,35]. Moreover, there is a possibility that patients are alone at home and unable to ask for assistance. However, some studies showed that stroke symptoms appearing either during the nighttime or daytime do not have a statistically significant association with PHD [11,15]. Stroke onset during the weekend increased the likelihood of early hospitalization in time for thrombectomy with no advanced neuroimaging (OR 2.64; 95% CI 1.19–5.85). Moreover, the unavailability of the primary care physician on the weekend may prompt patients to go directly to the hospital ED to avoid delays [18,27]; this should be promoted. If the prehospital recognition and transfer protocol (stroke code) were activated, patients arrived at the hospital sooner (OR 6.46; 95% CI 1.71–24.42) [18,36]. The same factor could be confusing because its activation is more frequent if the EMS is the first medical contact or if the patient arrives at the hospital on time for fibrinolysis or mechanical thrombectomy.

Public awareness campaigns on stroke have shown little success to date [37]. These campaigns must be updated and include the cognitive, contextual, and behavioral factors identified in this study, as well as the recent increase in the time window for reperfusion therapy [5,38]. Their main targets are not only at-risk patients, but also the general population, which can witness a stroke attack and should be aware of the risk with age.

This study had some limitations: It was performed at a third-level hospital, and additional analyses, including the indications of mechanical thrombectomy published in 2018, were not performed because the study had been completed prior to its publication. The strengths of this study were the consecutive, rather than retrospective, nature at the time of stroke and the detailed analysis of the PHD factors associated with the patients, some of which were not studied previously.

## 5. Conclusions

This study identified different contextual, cognitive, and behavioral factors that decrease the PHD and the time until reperfusion treatment. It is necessary to develop public awareness campaigns, wherein asking for help should be the first response after the onset of stroke symptoms. Telestroke and new neuroimaging techniques for reperfusion have improved the diagnosis and treatment of stroke occurring at nighttime or in rural areas. The activation of the prenotification system (stroke code) can help patients arrive at the hospital sooner; therefore, all health professionals must know when and how to use it.

## Figures and Tables

**Table 1:** Time from symptom onset to hospital arrival by socio-demographic factors.

Socio-Demographic Factors	*n* (%)	PHD, Minutes	*p*-Value
		Median (IQR)	
Age:			
≤75	118 (36.65)	126 (73–272)	0.334
>75	204 (63.35)	148 (75–356)	
Sex:			
Male	181 (56.21)	133 (79–293)	0.839
Female	141 (43.79)	152 (65–359)	
Marital status:			
Married	146 (45.34)	132.5 (84–272)	0.804
Not married at the moment	176 (54.66)	150 (70–344)	
Educational level:			
No or primary study	211 (65.53)	142 (75–327)	0.819
Secondary or higher study	111 (34.47)	129 (72–335)	
Average annual income:			
≤€20.000	239 (74.22)	140 (75–327)	0.753
>€20.000	83 (25.78)	128 (70–335)	
Living arrangements:			
Alone	70 (21.74)	190 (64–401)	0.511
With others	252 (78.26)	133 (75–299)	

PHD: prehospital delay; IQR: interquartile range.

**Table 2:** Time from symptom onset to hospital arrival by clinical factors.

Clinical Factors	n (%)	PHD, Minutes	*p*-Value
		Median (IQR)	
Arterial hypertension			
Yes	225 (69.88)	139 (75–332)	0.754
No	97 (30.12)	136 (73–305)	
Diabetes mellitus			
Yes	80 (24.84)	129.5 (70–310)	0.904
No	242 (75.16)	139 (74–335)	
Dyslipidemia			
Yes	158 (49.07)	129 (84–359)	0.754
No	164 (50.93)	151 (69–299)	
Overweight/obesity			
Yes	214 (66.46)	132.5 (75–327)	0.817
No	108 (33.54)	168 (70–333)	
Cardiovascular disease			
Yes	159 (49.38)	138 (75–359)	0.937
No	163 (50.62)	142 (74–309)	
Atrial fibrillation			
Yes	101 (31.37)	130 (69–374)	0.387
No	221 (68.63)	150 (80–305)	
Previous stroke			
Yes	73 (22.67)	115 (65–245)	0.078
No	249 (77.33)	146 (79–356)	
Family history of stroke			
Yes	114 (35.40)	148 (85–350)	0.210
No	208 (64.60)	132.5 (70–322)	
Active smoker			
Yes	52 (16.15)	130.5 (72–393)	0.864
No	270 (83.85)	140 (75–311)	
Alcohol consumption			
Yes	147 (45.65)	136 (80–335)	0.702
No	175 (54.35)	140 (70–294)	
Physical activity			
>3 days per week	148 (45,96)	136 (78–335)	0.648
<3 days per week	174 (54,04)	139 (71–295)	
Motor symptoms			
Yes	215 (66.77)	129 (67–294)	0.151
No	107 (33.23)	156 (95–336)	
Sensitive symptoms			
Yes	37 (11.49)	133 (68–399)	0.718
No	285 (88.51)	139 (75–314)	
Speech/language disturbance			
Yes	207 (64.29)	120 (67–249)	***0.001***
No	115 (35.71)	197 (92–411)	
Alteration of consciousness			
Yes	37 (11.49)	167 (74–342)	0.533
No	285 (88.51)	133 (74–323)	
Alteration of vision			
Yes	31 (9.63)	118 (61–286)	0.319
No	291 (90.37)	139 (75–335)	
Dizziness/instability			
Yes	72 (22.36)	134.5 (83–435)	0.182
No	250 (77.64)	139.5 (69–281)	
Headache			
Yes	41 (12.73)	170 (102–486)	***0.035***
No	281 (87.27)	133 (70–297)	
Previous similar symptoms			
Yes	82 (25,47)	125.5 (65–628)	0.200
No	240 (74.53)	145 (78–345)	
Onset mode of symptoms			
Gradual/stepwise	36 (11.19)	190.5 (104–381)	0.083
Sudden	286 (88.81)	133.5 (16–718)	
Type of stroke			
Ischemic	278 (86.34)	135 (73–328)	0.513
Hemorrhagic	44 (13.66)	158.5 (84–360)	
Affected cerebral hemisphere			
Right	121 (37.58)	144 (71–331)	0.921
Left	182 (56.52)	133 (75–333)	
Bilateral	19 (5.90)	184 (75–286)	
Stroke severity			
NIHSS ≤ 16	267 (82.92)	144 (74–335)	0.446
NIHSS > 16	55 (17.08)	111 (75–272)	
Previous level of dependence			
mRS > 2	246 (76.40)	141 (74–328)	0.826
mRS ≤ 2	76 (23.60)	135.5 (72–342)	

PHD: prehospital delay; IQR: interquartile range; NIHSS: National Institute of Health Stroke Scale; mRS: modified Rankin scale.

**Table 3:** Time from symptom onset to the hospital arrival by behavioral factors.

Behavioral Factors	n (%)	PHD, Minutes	*p*-Value
		Median (IQR)	
Type of coping			
Active	123 (38.20)	140 (75–273)	0.651
Passive	199 (61.80)	136 (72–359)	
First response after onset of symptoms:			
Asked for help	188 (58.39)	91.5 (56–135)	<0.001
Did not ask for help	134 (41.61)	289.5 (198–458)	
First medical contact			
EMS	143 (44.41)	106 (58–232)	<0.001
PCP	92 (28.57)	198 (110–382)	
Hospital	87 (27.02)	150 (72–397)	
Previous use of EMS:			0.714
Yes	186 (57.76)	132.5 (70–335)	
No	136 (42.24)	141 (80–307)	

PHD: prehospital delay; IQR: interquartile range; EMS: emergency medical service; PCP: primary care physician.

**Table 4:** Time from the symptom onset to hospital arrival by cognitive factors.

Cognitive Factors	*n* (%)	PHD, Minutes	*p*-Value
		Median (IQR)	
Symptoms attributed:			
Possibly stroke	82 (25.47)	95.5 (55–138)	<0.001
Possibly not stroke	240 (74.53)	195.5 (91–389)	
Self-perceived level of seriousness:			
Not serious (1–3)	259 (80.43)	169 (85–376)	<0.001
Serious (4–5)	63 (19.57)	98 (53–152)	
Anxiety level:			
Low (1–3)	216 (67.07)	185 (83–389)	<0.001
High (4–5)	106 (32.93)	108 (65–193)	
Thought, the situation could be self-managed:			
Yes	80 (24.85)	359 (247–537)	<0.001
No	242 (75.15)	106 (63–195)	
Thought, symptoms would improve:			
Yes	95 (29.50)	329 (193–485)	<0.001
No	227 (70.50)	106 (63–199)	
Previous knowledge of stroke:			
Yes	179 (55.59)	125 (71–265)	0.009
No	143 (44.41)	184 (84–425)	
Previous knowledge of stroke risk factors:			
Yes	168 (52.17)	125 (67–266)	0.011
No	154 (47.83)	184 (86–401)	
Previous knowledge of acting after a stroke:			
Yes	112 (34.78)	76 (50–151)	<0.001
No	210 (65.22)	196.5 (106–421)	

PHD: prehospital delay; IQR: interquartile range.

**Table 5:** Time from symptom onset to hospital arrival by contextual factors.

Contextual Factors	n (%)	PHD, Minutes	*p*-Value
		Median (IQR)	
Person who recognized symptoms:			
Patient	204 (63.35)	128 (71–296)	0.057
Witness	118 (36.65)	180 (83–397)	
Person who requested assistance:			
Patient	175 (54.35)	150 (75–376)	0.140
Witness	147 (45.65)	125 (71–267)	
Situation in which found:			
Alone	88 (27.33)	257.5 (117–446)	<0.001
Accompanied	234 (72.67)	115 (65–250)	
Time of day:			
Morning (06:00–14:00)	152 (47.20)	146.5 (72–335)	<0.001
Afternoon (14:00–22:00)	122 (37.89)	110 (65–210)	
Night (22:00–08:00)	48 (14.91)	289 (112–650)	
Type of day:			
Working day	219 (68.01)	142 (80–390)	0.093
Weekend	103 (31.99)	132 (67–251)	
Onset of symptoms at:			
Home	244 (75.76)	184 (91–396)	<0.001
Other localization	78 (24.24)	92.5 (55–104)	
Area:			
Urban	191 (59.32)	116 (56–359)	0.005
Rural	131 (40.68)	152 (105–309)	
Mode of arrival:			
Ambulance	221 (68.63)	128 (71–268)	0.046
Others	101 (31.37)	184 (86–404)	
Prehospital stroke code activated			
Yes	84 (26.09)	87 (58–135)	<0.001
No	238 (73.91)	196.5 (95–400)	

PHD: prehospital delay; IQR: interquartile range; EMS: emergency medical service.

**Table 6:** Multivariable regression analysis of independent predictors of delay ≤210 min and ≤360 min from the symptom onset to hospital arrival.

Factors	PHD ≤ 210 min		PHD ≤ 360 min	
	OR^a^ (95% CI)	*p*-Value	OR (95% CI)	*p*-Value
Speech/language disturbance: Yes	2.21 (1.16–4.36)	*0.023*	-	-
First response after onset of symptoms: Asked for help	10.36 (4.47–23.99)	*<0.001*	-	-
Symptoms attributed: Possibly stroke	-	*-*	1.98 (1.03–3.82)	0.041
Thought the situation could be self-managed: No	4.14 (1.70-10.07)	*0.002*	5.14 (2.60–10.16)	<0.001
Previous knowledge of acting after a stroke: Yes	-	*-*	3.20 (1.38–7.40)	0.007
Time of day: Day (08:00–22:00)	7.73 (3.09–19.34)	*<0.001*	3.79 (1.71–8.39)	0.001
Type of day: Weekend		*-*	2.64 (1.19–5.85)	0.017
Onset of symptoms at home: No	3.03 (1.22–7.55)	*0.017*	7.09 (1.97–25.55)	0.003
First medical contact: EMS	2.77 (1.32–5.88)	*0.008*		-
Prehospital stroke code activated: Yes	4.54 (1.77–11.64)	*0.002*	6.46 (1.71–24.42)	0.006

PHD: prehospital delay; OR: odds ratio; CI: confidence interval; EMS: emergency medical service. ^a^ Odds ratio greater than 1 indicates a positive association with delay ≤210 and ≤360 min.

## References

[1] Thrift A.G., Cadilhac D.A., Thayabaranathan T., Howard G., Howard V.J., Rothwell P.M., Donnan G.A. (2017). Global stroke statistics. Int. J. Stroke.

[2] Benjamin E.J., Blaha M.J., Chiuve S.E., Cushman M., Das S.R., Deo R., De Ferranti S.D., Floyd J., Fornage M., Gillespie C. (2017). Heart Disease and Stroke Statistics—2017 Update: A Report from the American Heart Association. Circulation.

[3] Johnson C.O. (2019). Global, regional, and national burden of stroke, 1990–2016: A systematic analysis for the Global Burden of Disease Study 2016. Lancet Neurol..

[4] Ahmed N., Wahlgren N., Grond M., Hennerici M., Lees K.R., Mikulik R., Parsons M., Roine R.O., Toni D., Ringleb P. (2010). Implementation and outcome of thrombolysis with alteplase 3-4,5 h after an acute stroke: An updated analysis from SITS-ISTR. Lancet Neurol..

[5] Albers G.W., Marks M.P., Kemp S., Christensen S., Tsai J.P., Ortega-Gutierrez S., McTaggart R.A., Torbey M.T., Kim-Tenser M., Leslie-Mazwi T. (2018). Thrombectomy for Stroke at 6 to 16 Hours with Selection by Perfusion Imaging. N. Engl. J. Med..

[6] Singer O.C., Hamann G.F., Misselwitz B., Steinmetz H., Foerch C. (2012). Time trends in systemic thrombolysis in a large hospital-based stroke registry. Cerebrovasc. Dis..

[7] Asaithambi G., Tong X., George M.G., Tsai A.W., Peacock J.M., Luepker R.V., Lakshminarayan K. (2014). Acute stroke reperfusion therapy trends in the expanded treatment window era. J. Stroke Cerebrovasc. Dis..

[8] Nasr D.M., Brinjikji W., Cloft H.J., Rabinstein A.A. (2013). Utilization of intravenous thrombolysis is increasing in the United States. Int. J. Stroke.

[9] Hoffmeister L., Lavados P.M., Mar J., Comas M., Arrospide A., Castells X. (2016). Minimum intravenous thrombolysis utilization rates in acute ischemic stroke to achieve population effects on disability: A discrete-event simulation model. J. Neurol. Sci..

[10] Powers W., Rabinstein A., Ackerson T., Adevoe O., Bambakidis N., Becker K. (2018). 2018 Guidelines for the Early Management of Patients with Acute Ischemic Stroke: A Guideline for Healthcare Professionals From the American Heart Association/American Stroke Association. J. Vasc. Surg..

[11] Hong E.S., Kim S.H., Kim W.Y., Ahn R., Hong J.S. (2011). Factors associated with prehospital delay in acute stroke. Emerg. Med. J..

[12] Faiz K.W., Sundseth A., Thommessen B., Rønning O.M. (2013). Prehospital delay in acute stroke and TIA. Emerg. Med. J..

[13] Madsen T.E., Sucharew H., Katz B., Alwell K.A., Moomaw C.J., Kissela B.M., Flaherty M.L., Woo D., Khatri P., Ferioli S. (2016). Gender and time to arrival among ischemic stroke subjects in the Greater Cincinnati/Northern Kentucky Stroke Study. J. Stroke Cerebrovasc. Dis..

[14] Sim J., Shin C.-N., An K., Todd M. (2016). Factors Associated with the Hospital Arrival Time in Patients with Ischemic Stroke in Korea. J. Cardiovasc. Nurs..

[15] Mandelzweig L., Goldbourt U., Boyko V., Tanne D. (2006). Perceptual, social, and behavioral factors associated with delays in seeking medical care in subjects with symptoms of acute stroke. Stroke.

[16] Montaner J., Alvarez-Sabín J. (2006). NIH stroke scale and its adaptation to Spanish. Neurología.

[17] Morán C., Landero R., González M.C.T. (2010). COPE-28: Un análisis psicométrico de la versión en Español del brief COPE. Univ. Psychol..

[18] Ruiz R.G., Fernández J.S., Ruiz R.M.G., Bermejo M.R., Arias Á.A., del Saz Saucedo P., Arroyo R.H., Manero A.G., Pinto A.S., Muñoz S.N. (2017). Response to symptoms and prehospital delay in stroke subjects. Is it time to reconsider stroke awareness campaigns?. J. Stroke Cerebrovasc. Dis..

[19] Geffner D., Soriano C., Pérez T., Vilar C., Rodríguez D. (2011). Delay in seeking treatment by subjects with stroke: Who decides, where they go, and how long it takes. Clin. Neurol. Neurosurg..

[20] Herkes G., Barr J., McKinley S., O’Brien E. (2006). Patient Recognition of and Response to Symptoms of TIA or Stroke. Neuroepidemiology.

[21] Kim Y.S., Park S.-S., Bae H.-J., Cho A.-H., Cho Y.-J., Han M.-K., Heo J.H., Kang K., Kim D.-E., Kim H.Y. (2011). Stroke awareness decreases prehospital delay after acute ischemic stroke in korea. BMC Neurol..

[22] Zhou Y., Yang T., Gong Y., Li W., Chen Y., Li J., Wang M., Yin X., Hu B., Lu Z. (2017). Pre-hospital delay after acute ischemic stroke in central urban China: Prevalence and risk factors. Mol. Neurobiol..

[23] Goyal M., Demchuk A.M., Menon B.K., Eesa M., Rempel J.L., Thornton J., Roy D., Jovin T.G., Willinsky R.A., Sapkota B.L. (2015). Randomized Assessment of Rapid Endovascular Treatment of Ischemic Stroke. N. Engl. J. Med..

[24] Jansen I.G.H., Mulder M.J.H.L., Goldhoorn R.J.B. (2018). Endovascular treatment for acute ischaemic stroke in routine clinical practice: Prospective, observational cohort study (MR CLEAN Registry). BMJ.

[25] Hand P., Kwan J., Sandercock P. (2004). A systematic review of barriers to delivery of thrombolysis for acute stroke. Age Ageing.

[26] Jiang B., Ru X., Sun H., Liu H., Sun D., Liu Y., Huang J., He L., Wang W. (2016). Pre-hospital delay and its associated factors in first-ever stroke registered in communities from three cities in China. Sci. Rep..

[27] Palomeras E., Fossas P., Quintana M., Monteis R., Sebastian M., Fábregas C., Ciurana A., Ribó M., Cano A., Sanz P. (2008). Emergency perception and other variables associated with extra-hospital delay in stroke subjects in the Maresme region (Spain). Eur. J. Neurol..

[28] Moloczij N., McPherson K.M., Smith J.F., Kayes N.M. (2008). Help-seeking at the time of stroke: Stroke survivors’ perspectives on their decisions. Health Soc. Care Community.

[29] Eissa A., Krass I., Levi C., Sturm J., Ibrahim R., Bajorek B. (2013). Understanding the reasons behind the low utilisation of thrombolysis in stroke. Australas. Med. J..

[30] Papapanagiotou P., Iacovidou N., Spengos K., Xanthos T., Zaganas I., Aggelina A., Alegakis A., Vemmos K. (2011). Temporal trends and associated factors for pre-hospital and in-hospital delays of stroke subjects over a 16-year period: The Athens study. Cerebrovasc. Dis..

[31] Räty S., Silvennoinen K., Tatlisumak T. (2018). Prehospital pathways of occipital stroke subjects with mainly visual symptoms. Acta Neurol. Scand..

[32] Ragoschke-Schumm A., Walter S., Haass A., Balucani C., Lesmeister M., Nasreldein A., Sarlon L., Bachhuber A., Licina T., Grunwald I.Q. (2014). Translation of the ‘time is brain’ concept into clinical practice: Focus on prehospital stroke management. Int. J. Stroke.

[33] Hsieh M.J., Tang S.C., Chiang W.C., Huang K.Y., Chang A.M., Ko P.C.I., Tsai L.K., Jeng J.S., Ma M.H.M. (2014). Utilization of emergency medical service increases chance of thrombolytic therapy in patients with acute ischemic stroke. J. Formos. Med. Assoc..

[34] Turin T.C., Kita Y., Rumana N., Takashima N., Ichikawa M., Sugihara H., Morita Y., Miura K., Okayama A., Nakamura Y. (2009). The Time Interval Window between Stroke Onset and Hospitalization and Its Related Factors. Neuroepidemiology.

[35] Korkmaz T., Ersoy G., Kutluk K., Erbil B., Karbek Akarca F., Sönmez N., Demir Ö.F. (2010). An evaluation of pre-admission factors affecting the admission time of subjects with stroke symptoms. Turk. J. Emerg. Med..

[36] Puolakka T., Strbian D., Harve H., Kuisma M., Lindsberg P.J. (2016). Prehospital Phase of the Stroke Chain of Survival: A Prospective Observational Study. J. Am. Heart Assoc..

[37] Lecouturier J., Rodgers H., Murtagh M.J., White M., Ford G.A., Thomson R.G. (2010). Systematic review of mass media interventions designed to improve public recognition of stroke symptoms, emergency response and early treatment. BMC Public Health.

[38] Nogueira R.G., Jadhav A.P., Haussen D.C., Bonafé A., Budzik R.F., Bhuva P., Yavagal D.R., Ribo M., Cognard C., Hanel R.A. (2018). Thrombectomy 6 to 24 Hours after Stroke with a Mismatch between Deficit and Infarct. N. Engl. J. Med..

